# New Perspectives on Thiamine Catalysis: From Enzymic to Biomimetic Catalysis

**DOI:** 10.1155/2007/23286

**Published:** 2007-04-17

**Authors:** A. Stamatis, G. Malandrinos, M. Louloudi, N. Hadjiliadis

**Affiliations:** Department of Chemistry, University of Ioannina, 45110 Ioannina, Greece

## Abstract

This paper is a brief review of the detailed mechanism of action of thiamine enzymes, based on metal complexes of bivalent transition and post-transition metals of model compounds, thiamine derivatives, synthesized and characterized with spectroscopic techniques and X-ray crystal structure determinations. It is proposed that the enzymatic reaction is initiated with a V conformation of thiamine pyrophosphate, imposed by the enzymic environment. Thiamine pyrophosphate is linked with the proteinic substrate through its pyrophosphate oxygens. In the course of the reaction, the formation of the “active aldehyde” intermediate imposes the S conformation to thiamine, while a bivalent metal ion may be linked through the N1' site of the molecule, at this stage. Finally, the immobilization of thiamine and derivatives on silica has a dramatic effect on the decarboxylation of pyruvic acid, reducing the time of its conversion to acetaldehyde from 330 minutes for the homogeneous system to less than 5 minutes in the heterogenous system.

## 1. INTRODUCTION

**Scheme 1:**
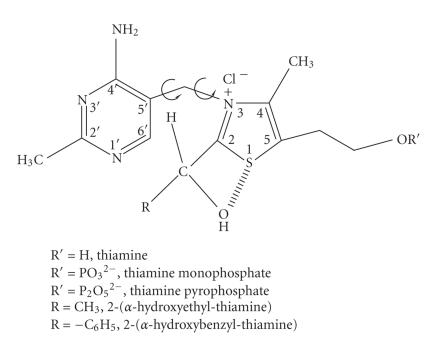


Thiamine pyrophosphate (TPP) is the cofactor of many enzymes, including carboxylase, transketolase, phosphoketolase, and so forth (see Scheme 1) [1]. It catalyzes the decarboxylation of *α*-ketoacids and the formation of *α*-ketols.

Thiamine pyrophosphate can undertake three conformations, depending on the relative orientations of the two rings of pyrimidine and thiazolium, determined by the torsional angles Φ_T_ = C(5′)–C(3, 5′)–N(3)–C(2), Φ_P_ = N(3)–C(3, 5′)–C(5′)–C(4′). These conformations are the common F, found in all derivatives of free thiamine, with Φ_T_ = 0° and Φ_P_ = ±90; the S conformation, constantly found in all 2-substituted derivatives of thiamine, with Φ_T_ = ±100° and Φ_P_ = ±150°, and the most rare V conformation with Φ_T_ = ±90° and Φ_P_ = ±90°, where the C′_4_–NH_2_ group approaches the C_2_–H of thiazolium [2].

Bivalent metals are also required for the action of thiamine enzymes (e.g., Mg^2+^, Ca^2+^ in vivo) or transition or post-transition metals (e.g., Ni^2+^, Co^2+^, Zn^2+^, Cd^2+^, etc., in vitro).

**Scheme 2:**
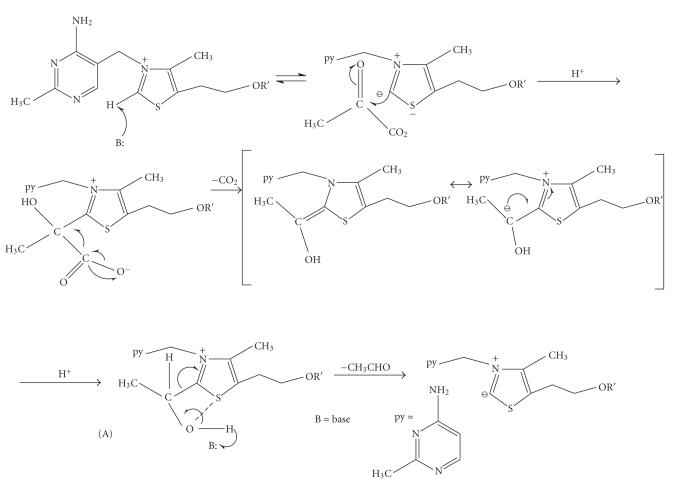


The accepted mechanism of action of thiamine in its enzymes was proposed by Breslow [3] and involves the addition of pyruvic acid to the C_2_ atom of thiazolium, following its deprotonation and the ylide formation (see Scheme 2).

Intermediate (A) is called an “active aldehyde” and it presents an O^−^–S^+^ electrostatic interaction, contributing to the internal neutralization of the charge of thiazolium. Such “active aldehyde” intermediates can be isolated [1].

Despite the general acceptance of Breslow mechanism, there remain the following questions unanswered in it:

1. the role of bivalent metal ions, both in vivo (Mg^2+^) or in vitro (Mg^2+^, Co^2+^, Zn^2+^, Ni^2+^, Cd^2+^, etc.);
2. the role of various parts and the conformation taken by TPP during the various enzymic steps.

We have attempted to provide answers to these questions and proposed a detailed mechanism of action by studying the interactions of bivalent metals, with thiamine model compounds, in recent years [1, 4]. We have subsequently tried to immobilize thiamine and derivatives into silica and look upon the catalytic properties of the composite materials produced.

In this paper, we are briefly reviewing all these efforts and conclusions drawn and proposing future applications.

## 2. INTERACTION OF THIAMINE WITH BIVALENT METAL IONS

**Scheme 3:**
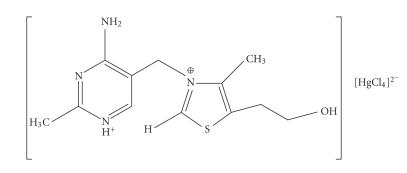


To investigate the role of bivalent metals in the enzymatic action of thiamine, it is important to elucidate the way the metals are bound to thiamine derivatives, such as coordination sites. Towards this goal, early research was directed in the preparation and elucidation of structures of bivalent metals with thiamine and derivatives. However, all these failed to give an answer to this question, due to the net positive charge on thiazolium ring and the easy protonation of N′_1_ atom of pyrimidine (pKa ≅ 5) resulting in double charged species. Thus, several double salt-type complexes of thiamine were reported in literature [1] of formulae [MX_4_]^2−^ [Th]^2+^, [MX_4_]^2−^ [Th]_2_^+^, or [MX_3_]_2_^−^[Th]^2+^, depending on pH. (M is Co^2+^, Ni^2+^, Zn^2+^, etc.; X is Cl^−^, Br^−^; Th is thiamine). All these contained no direct metal-ligand bonding. An example is the Hg^2+^ complex [5] of the formula in Scheme 3.

**Scheme 4:**
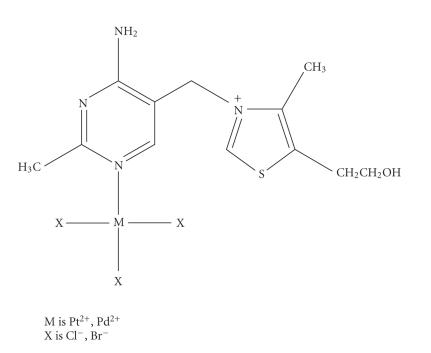


The earlier reported Pt^2+^ and Pd^2+^ thiamine derivatives (Scheme 4), however, were for the first time proposed to have a zwitterionic structure, with the metals bound at the N′_1_ site of pyrimidine [6]. This demonstrated the importance of this position as potential coordination site of metal ions. It indicated also that the net positive charge on N_3_ of thiazolium was the real reason for the difficulty to form complexes with direct metal-ligand bonds. The proposed structures were later confirmed by X-ray crystal structure determinations for these and other bivalent metals [1].

In the “active aldehyde” intermediates of thiamine catalysis (Scheme 2), the net positive charge on N_3_ of thiazolium is partially internally neutralized, due to the S^+^–O^−^ electrostatic interaction, resulting by a partial positive charge migration to the S_1_ position of the ring. We therefore thought that if “active aldehyde” derivatives of thiamine were used, instead of thiamine itself, the problem of complex formation with direct metal-ligand bonds might be overcome. This was proven to be true, using the “active aldehydes” 2-(*α*-hydroxybenzyl)thiamine (HBT) and 2-(*α*-hydroxycyclohexylmethyl)thiamine (HCMT) and the metals Zn^2+^, Cd^2+^, Hg^2+^, Co^2+^, and Ni^2+^ [7, 8]. Working at pH ∼ 5.3, all complexes obtained corresponded to the zwitterionic formulae MLCl_3_ and presented an M-N′_1_ direct interaction [7, 8]. Important was also the fact that all ligands either free or complexed were adopting the S conformation, and that the internal S^+^–O^−^ interaction continued to be the same in the complexes, like the ligands.

These findings led to the following conclusions:

1. the easy formation of complexes with direct metal-ligand bonds of bivalent metals with thiamine “active aldehyde” derivatives, than thiamine itself, strongly indicates that the metal interaction in the enzymatic action should follow the formation of the “active aldehyde” intermediates.
2. The S conformation may be important in the enzymatic cycle, since it is retained after the formation of the direct metal-ligand bonds.

## 3. INTERACTION OF THIAMINE MONO- AND PYROPHOSPHATE “ACTIVE ALDEHYDE” DERIVATIVES WITH BIVALENT METAL IONS

Since the phosphate esters of thiamine are the factors for its enzymatic action, a better conclusion on the role of metal ions cannot be drawn without a detailed study of the interaction of bivalent metals with thiamine phosphate esters. With this aim we continued our efforts starting with the interaction of “active aldehyde” derivatives of thiamine monophosphate (HBTMP) with bivalent metal ions, followed by “active aldehyde” derivatives of thiamine pyrophosphate (HBTPP and HETPP).

Depending on pH, three types of complexes were obtained from the interaction of HBTMP with Zn^2+^, Cd^2+^, and Hg^2+^ in aqueous solutions. There exist at pH ∼ 1 the double salts of formula [MCl_4_]^2−^ [LH]_2_^2+^ (L is HBTMP; and M are the named metals), at pH ∼ 3.5 the M(LH)Cl_3_ of a zwitterionic formula, with the metals bonded through the phosphate moiety, and at pH ∼ 6 complexes of formula MLCl_2_ [9], with the metals simultaneously bonded through N′_1_ and pyrophosphate oxygens [9]. Hg^2+^ produced the complex HgL_2_Cl_2_, at pH ∼ 6 with the metal bound at the N′_1_ site of the pyrimidine moiety only. The crystal structure of the free ligand and the HgL_2_Cl_2_ complex showed an S conformation for the ligand [9]. ^1^H NMR ROESY spectra confirmed the existence of the S conformation for the free ligand in solution, in all cases, demonstrated by a cross peak between the C_4_–CH_3_ group of thiazolium and the C′_6_–H of pyrimidine [10].

Using HETPP and HBTPP as ligands (L), we also prepared complexes of formulae {K[Zn(LH)Cl_2_(H_2_O)]}_m_, {K[Cd(LH)Cl_2_(H_2_O)]}_m_, K_2_[Hg(LH)_2_Cl_2_], and Zn(LH)_2_Cl_2_. Both sites of pyrimidine and pyrophosphate oxygens are once more shown to be potential coordination sites [10, 11]. The X-ray crystal structure determination of the ligand has again shown the S conformation for it [10]. The same conformation was adopted by the ligand in all isolated complexes, as the ^1^H NMR ROESY spectra revealed [10, 11].

These studies led to the following conclusions:

1. both the N′_1_ site and the phosphate group of thiamine phosphates may be important metal binding sites;
2. the importance of the S conformation in the mechanism of the enzymatic action of thiamine is emphasized;
3. the nature of the metal is important in determining the coordination site. For example, in contrast to the lighter Zn^2+^, Cd^2+^ metals, the heavier Hg^2+^ reacts only with N′_1_ site of thiamine monophosphate, even at pH ∼ 6.

## 4. THE CONFORMATION THAT THIAMINE MAY UNDERTAKE DURING THE ENZYMATIC ACTION

Using the model peptide Asp·Asp·Asn·Lys·Ileu mimicking the protein environment, surrounding the pyrophosphate moiety of TPP, we studied the ternary system [M^2+^]-[peptide]-[HETPP] of Zn^2+^, Cd^2+^, and Cu^2+^ [12, 13]. The pyrophosphate oxygen atoms and the N′_1_ site of pyrimidine were once more proven to be the metal coordination sites and thiamine once more was found in the S conformation.

It should be noted here that the base approaching the C_2_–H atom of thiazole was earlier proposed to be the 4′-NH_2_ group of pyrimidine, thereby forming the ylide, by attracting its proton and initiating the reaction [14]. Such a role of the 4′-NH_2_ group requires a V conformation for thiamine, found only rarely in its free derivatives. However, this conformation was constantly found in the crystal structure of several thiamine examples, obviously imposed and stabilized by the enzymic environment [15, 16]. Metals like Mg^2+^ and Ca^2+^ were linking thiamine pyrophosphate with the apoenzyme through the pyrophosphate group [15, 16].

**Scheme 5:**
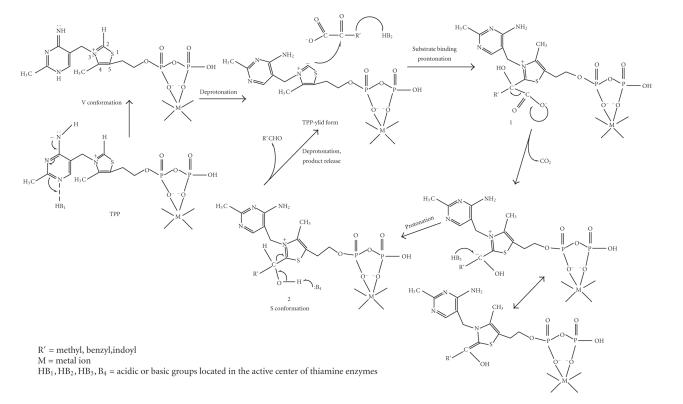


These results showed that both the V and S conformations are important and possibly succeed each other in the mechanism of action of thiamine enzymes [4, 17], as shown in Scheme 5. The V conformation initiates the reaction by attracting a proton from C_2_ and creating an ylide, and the S conformation is adopted after the formation of the “active aldehyde” intermediates, when a metal may also coordinate with N′_1_, besides the pyrophosphate group. The S conformation favors an S^+^–O(2a)^−^ electrostatic interaction, which facilitates the release of a proton and aldehyde and regenerates the ylide.

## 5. BIOMIMETIC CATALYSIS

The tethering of thiamine pyrophosphate and its derivatives on silica *via* the pyrophosphate group is expected to produce novel composite biomimetic materials, where silica would replace the protein environment of the natural enzyme. These materials would also be expected to catalyze thiamine reactions in vitro.

**Scheme 6:**
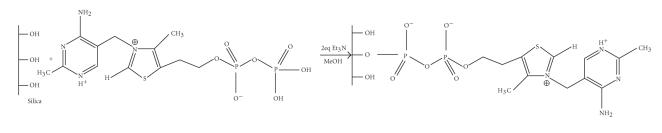


In fact, TPP and its derivatives were immobilized on silica, [18, 19] according to the reaction in Scheme 6.

In the same way, the “active aldehyde” intermediates HBTPP and HETPP were also immobilized on silica and their catalytic properties were evaluated and compared with the ones of TPP itself. The materials obtained corresponded to formulae [HBTPP–OP_2_O_6_–SiO_3/2_]_n_ · xSiO_2_, [HET–OP_2_O_6_–SiO_3/2_]xSiO_2_, and [Th–OP_2_O_6_–SiO_3/2_]_n_ · xH_2_O. Their catalytic activities were subsequently evaluated in the absence of the corresponding aldehydes (1), (3) or in the presence of them (2), (4), as shown below:

(1) $$2{\text{CH}}_{\text{3}}{\text{COCOO}}^{-}\overset{\text{thiamin}}{\underset{\text{catalyst}}{\to }}{\text{2CO}}_{\text{2}}+{\text{H}}_{\text{3}}{\text{CCOCH(OH)CH}}_{\text{3}}\text{,}\;\;\;\;\text{(acetoine)}$$

(2) $${\text{CH}}_{\text{3}}{\text{COCOO}}^{-}+{\text{CH}}_{\text{3}}\text{CHO }\overset{\text{thiamin}}{\underset{\text{catalyst}}{\to }}{\text{CO}}_{\text{2}}+{\text{H}}_{\text{3}}{\text{CCOCH(OH)CH}}_{\text{3}}\text{,}\;\;\;\;\text{(acetoine)}$$

(3) $${\text{2C}}_{\text{6}}{\text{H}}_{\text{5}}{\text{COCOO}}^{-}\overset{\text{thiamin}}{\underset{\text{catalyst}}{\to }}{\text{2CO}}_{\text{2}}+{\text{C}}_{\text{6}}{\text{H}}_{\text{5}}{\text{COCH(OH)C}}_{\text{6}}{\text{H}}_{\text{5}}\text{,}\;\;\;\;\text{(benzoin)}$$

(4) $${\text{C}}_{\text{6}}{\text{H}}_{\text{5}}{\text{COCOO}}^{-}+{\text{C}}_{\text{6}}{\text{H}}_{\text{5}}\text{CHO}\overset{\text{thiamin}}{\underset{\text{catalyst}}{\to }}{\text{CO}}_{\text{2}}+{\text{C}}_{\text{6}}{\text{H}}_{\text{5}}{\text{COCH(OH)C}}_{\text{6}}{\text{H}}_{\text{5}},\;\;\;\;\text{(benzoin).}$$

The results on benzoyl-formate decarboxylation are summarized in Table 1 and compared with the homogeneous systems.

**Scheme 7:**
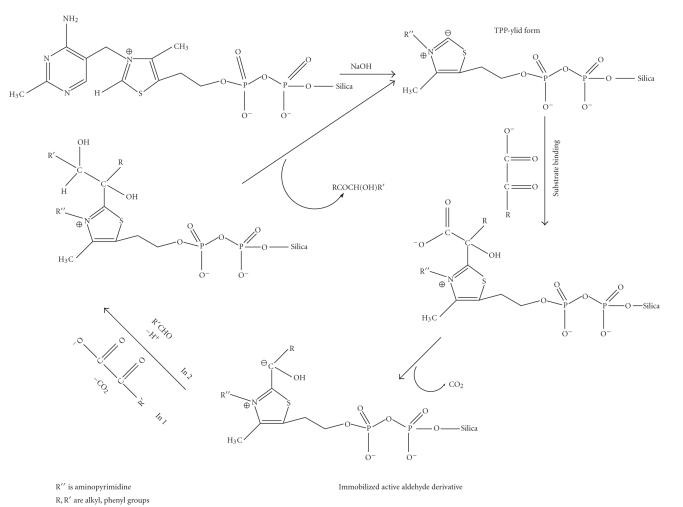


It is thus obvious that the immobilized thiamine pyrophosphate derivatives are far more efficient catalysts than the corresponding homogeneous systems, reducing the reaction time from 330 minutes of the homogeneous system to less than 5 minutes only, for 100% yield in both reactions (1), (3) and (2), (4). Detailed mechanisms of action have been proposed for both reactions as shown in Scheme 7.

These very effective and promising heterogeneous biocatalysts may be evaluated in future studies for the enantioselective production of optically active 2-hydroxy-ketones.

## Figures and Tables

**Table 1:** Benzoyl-formate decarboxylation catalyzed by thiamine catalysts. (1) Reaction conditions: all reactions were carried out at 37°C in MeOH (1 mL) with benzoyl formate (200 *μ*mol), thiamine catalyst (20 *μ*mol), and NaOH (40 *μ*mol). (2) Reaction conditions: all reactions were carried out at 37°C in MeOH (1 mL) with benzoyl formate (200 *μ*mol), benzaldehyde (400 *μ*mol), thiamine catalyst (20 *μ*mol), and NaOH (40 *μ*mol). In both cases, bromo benzene was used as an internal standard.

Catalyst	Reaction time(min)	Conversion (%)

TPP (homogeneous system)	330	82(1)
HBTPP (homogeneous system)	215	90(1)
HETPP (homogeneous system)	330	85(1)
[Th–OP_2_O_6_–SiO_3/2_]_n_· xSiO_2_	<5	100(1)
[HBT–OP_2_O_6_–SiO_3/2_]_n_· xSiO_2_	<5	100(1)
[HET–OP_2_O_6_–SiO_3/2_]_n_· xSiO_2_	<5	100(1)
TPP (homogeneous system)	330	67(2)
HBTPP (homogeneous system)	250	74(2)
HETPP (homogeneous system)	330	72(2)
[Th–OP_2_O_6_–SiO_3/2_]_n_ · xSiO_2_	<5	100(2)
[HBT–OP_2_O_6_–SiO_3/2_]_n_ · xSiO_2_	<5	100(2)
[HET–OP_2_O_6_–SiO_3/2_]_n_ · xSiO_2_	<5	100(2)
